# Preparing medical students to incorporate scientific evidence into patient care: A cross-sectional study

**DOI:** 10.1371/journal.pone.0321211

**Published:** 2025-04-04

**Authors:** Susanna M. Wallerstedt, Martin Garwicz, Pontus Henriksson, Karin Mossberg, Estelle Naumburg, Jeanette Wahlberg, Riitta Möller

**Affiliations:** 1 Department of Pharmacology, Sahlgrenska Academy, University of Gothenburg, Gothenburg, Sweden; 2 HTA-centrum, Sahlgrenska University Hospital, Gothenburg, Sweden; 3 Department of Experimental Medical Science, Lund University, Lund, Sweden; 4 Department of Health, Medicine and Caring Sciences, Linköping University, Linköping, Sweden; 5 Department of Public Health and Community Medicine, Sahlgrenska Academy, University of Gothenburg, Sweden; 6 Närhälsan Herrestad Vårdcentral, Primary Health Care, Region Västra Götaland, Sweden; 7 Department of Clinical Sciences, Unit of Paediatrics, Umeå University, Umeå, Sweden; 8 School of Medical Sciences, Faculty of Medicine and Health, Örebro University, Örebro, Sweden; 9 Department of Endocrinology and Diabetes, Örebro University Hospital, Örebro, Sweden; 10 Department of Medical Epidemiology and Biostatistics, Karolinska Institutet, Stockholm, Sweden; 11 Department of Otolaryngology, Head and Neck Surgery, Karolinska University Hospital, Stockholm, Sweden; Birjand University of Medical Sciences, IRAN, ISLAMIC REPUBLIC OF

## Abstract

**Objective:**

To explore teaching- and assessment-related factors that predict medical students’ perceived attainment of sufficient skills to incorporate scientific evidence into patient care.

**Methods:**

An anonymous questionnaire was distributed to final-semester students in six medical programs in Sweden. The students were asked to rate statements concerning the extent to which 11 national degree outcomes related to the scientific basis of medicine (scholarly degree outcomes) had been adequately assessed during the program; their perceived preparedness for evidence-based patient care; and training during the program regarding the components of a systematic review/health technology assessment (HTA).

**Results:**

In total, 433 students (median age: 25 years [interquartile range: 24‒28], 59% female) participated in the study (response rate: 68%). A multivariate analysis indicated that experienced adequate assessment on a single scholarly degree outcome (i.e., “Demonstrate knowledge of the scientific foundation of medicine and insight into current research as well as knowledge of the link between science and proven experience”) predicted the students’ perception of having developed sufficient skills in incorporating scientific evidence into patient care (odds ratio: 6.17 [95% confidence interval: 3.10; 12.3]). The educational content predictors of this perception included the teaching of HTA (11.3 [1.44; 89.5]) and training regarding two components of a systematic review/HTA: appraising scientific articles using checklists (2.46 [1.23; 4.90]) and assessing organizational aspects related to the introduction/withdrawal of a health technology (2.65 [1.05; 6.67]). The presence of hands-on, credit-bearing, evidence-based medicine (EBM)-related learning activities during clinical courses was also predictive (4.68 [1.69; 13.0]).

**Conclusions:**

This study highlights important educational activities that prepare medical students to incorporate scientific evidence into patient care: (i) adequate assessment of key content regarding scholarly outcomes, including the scientific foundation of medicine; (ii) learning activities about HTA and the systematic review process; and (iii) hands-on application of EBM-related learning activities integrated into clinical courses.

## Introduction

As medical knowledge and technological advancements continue to grow, staying abreast will continue to be a key challenge in the practice of medicine. To address this challenge in medical education, evidence-based medicine (EBM) and health technology assessment (HTA) have emerged as crucial frameworks [1-3], supported by advancements in health informatics [4]. They inform decision-making in healthcare through systematic evidence syntheses that focus on the effectiveness, safety, and cost-effectiveness of interventions [5,6]. Fundamentally, EBM has been defined as the integration of (i) current best evidence assessed with a systematic approach, (ii) clinical expertise, and (iii) patient values in healthcare delivery [6]. Thus, EBM primarily concerns patient-level decisions. An HTA, by contrast, primarily provides information for healthcare decision-making above the individual patient level, that is, at various organizational levels. Thus, compilations of economic, societal, and ethical implications may constitute important additional components of an HTA [5,7].

Medical programs should include all steps of evidence-based practice in their curricula. This includes translating uncertainty into answerable questions, searching for and retrieving relevant evidence, critically appraising such evidence regarding validity and clinical importance, applying it to practice, and evaluating performance [8]. EBM, which originally focused on critical appraisal, the development of systematic reviews, and clinical practice guidelines, has also been described as essential in the training of young clinicians [9]. However, a recent systematic review reported that evidence regarding effective teaching modalities remains scant [10]. Additionally, research on the transfer of EBM skills to clinical practice is reportedly scarce [11]. Furthermore, a scoping review showed that many studies have focused on the education of other healthcare professionals, such as nursing students, rather than physicians [12].

Medical students in Sweden must meet 23 national outcomes to obtain a medical degree, as outlined in the Swedish Higher Education Ordinance. Of these 23 outcomes, 11 are related to scientific skills and attitudes (hereafter described as scholarly degree outcomes). This national framework plays a crucial role in promoting quality, consistency, and accountability, ensuring that graduates meet the needs of patients and healthcare systems [13]. However, this framework also allows universities to develop their own teaching practices, intending to support students in attaining learning outcomes. Previous research has shown that there are considerable differences among outcome frameworks related to scientific research —also called scholarly activities—between countries and, consequently, between teaching and assessment [14,15]. In this context, a better understanding of medical students’ experiences of educational content that has contributed to their perceived skills in evidence-based practice could be helpful. Therefore, the present study aimed to explore how final-semester medical students experience the assessment of scholarly degree outcomes and teaching and learning activities related to systematic reviews/HTA. Furthermore, we investigated the association between this experience and the students’ perceptions of having developed sufficient skills to incorporate scientific evidence into patient care.

## Methods

This cross-sectional study, which involved six medical programs in Sweden, was based on anonymous questionnaire responses from final-semester medical students and an overall mapping of scientifically related teaching and learning activities within the participating medical programs. The study was assessed by the Swedish Ethical Review Authority, which determined that the Ethical Review Act was not applicable and had no ethical objections to the study (2023-03120-0). Participation was voluntary, and the participants were informed about the study orally and in writing. Providing a completed questionnaire represented consent to participate. The recruitment period was September 9, 2023 to January 15, 2024.

### Setting

All seven medical programs in Sweden were invited to participate, and all but one accepted the invitation. Thus, the study was conducted within the context of six medical programs (University of Gothenburg, Karolinska Institute, Linköping University, Lund University, Umeå University, and Örebro University). All universities were currently in the process of implementing a six-year medical program, but the present study concerned the preceding 5.5-year program, including 2 to 2.5 years of primarily preclinical aspects related to medical science, and the remaining years mainly covering clinical education, including direct patient care. All programs integrated clinical activities early on. They also integrated the teaching of professionalism and scientific education throughout the program. Unlike the six-year medical program, about 1.5 years of internship was required after the 5.5-year program to obtain a medical certificate. However, medical students who passed the 9^th^ semester could obtain a temporary appointment to work within the profession.

During the 4th or 5th year, all the programs incorporated a mandatory research project course that spanned approximately 20 weeks and corresponded to 30 European Credit Transfer System (ECTS) credits. During this course, each student conducted an individual research project and presented a research report that was structured akin to a scientific publication. Supervisors were active researchers, holding a minimum of a PhD, who could offer a suitable project within their area of expertise. Optional co-supervisor(s), such as PhD students or other researchers in the same area, could also be appointed.

Swedish medical programs, however, differ from each other in terms of the broader context of scientific education. Since course content and related learning outcomes are developed locally, it is not feasible to comprehensively map teaching and learning activities related to EBM. Nevertheless, two notable differences emerged when we mapped the number of ECTS credits for mandatory research projects as well as the presence and type of education specifically targeting EBM, either as a single course or longitudinally during clinical semesters. First, some programs offered hands-on, credit-bearing, EBM-related learning activities during clinical courses at the later stages of the medical program (Linköping and Lund), whereas others did not. In Linköping, this learning activity spanned the 6^th^, 7^th^, 9^th^, 10^th^, and 11^th^ semesters, comprising 1 ECTS per semester. In Lund, this learning activity spanned the 6^th^, 7^th^, and 8^th^ semesters, comprising 1.5 ECTS per semester. Second, some programs included an additional mandatory research project course (Lund and Örebro, in semesters 5 and 6, respectively), whereas the others did not. This educational element encompassed a scholarly project of 10 weeks (15 ECTS credits) that students carried out under supervision, either alone or in pairs.

### Participants

Medical students from the 11^th^ semester in six medical schools were invited to participate. A total of 642 students were registered for this semester at the time of the study. The students were informed orally and in writing about the aims of the study, stating that participation was voluntary and that opting out of participation would not affect their education. Printed copies of the questionnaire were distributed by the authors on an occasion scheduled adjacent to a routine mandatory learning activity during a curricular course. By answering the questionnaire, the students gave their informed consent to participate.

### Data collection

The data were retrieved using an anonymous questionnaire developed specifically for this study (see S1 File). It contained eight sections (45 items) in the following order: (I) experience regarding the adequacy of the assessment during the medical program in terms of the 11 scholarly degree outcomes (from a total of 23 degree outcomes) (11 items: 1a‒k), (II) the comprehensibility of these degree outcomes (11 items: 2a‒k); (III) perceptions regarding the attainment of sufficient skills in how to ground patient work on scientific evidence during the medical program (1 item: 3); (IV) experience of having undergone education on HTA during the program (1 item: 4); (V) experience of training during the medical program in scientific skills related to the procedural steps of a systematic review (5 items: 5a‒e) as well as an HTA based on a systematic review of patient benefits and harms of an intervention (8 items: 5a‒e, 6a‒c); (VI) knowledge-based single best answer (SBA) questions (5 items: 7‒11); (VII) open-ended questions (2 items: 12-13; not reported herein); and (VIII) responder characteristics (6 items: 14‒19). In Sections I‒V, the item statements were rated on a 5-point Likert scale from 1 (totally disagree) to 5 (totally agree). In Section VI, there were five SBAs with five response alternatives. These questions concerned the interpretation of research results of direct relevance for medical practice, that is, how to interpret (i) a case/control study, (ii) a diagnostic study, (iii) a meta-analysis with certainty of evidence expressed according to Grading of Recommendations, Assessment, Development, and Evaluations (GRADE) [16], (iv) a forest plot including a meta-analysis, and (v) a health economics statement regarding cost-effectiveness. The open-ended questions in Section VI (not reported in this article) asked the students about learning activities that had been useful for their development of skills to incorporate scientific evidence into patient care. Finally, responder characteristics (Section VII) included gender, age, previous doctoral degree, previous studies in other medical faculties, whether the 30-credit research project comprised a systematic review, and working life experience as an intern/junior doctor. It took approximately 15–20 minutes to complete the questionnaire. Local conditions determined whether the students could be compensated for their participation, for example, with a sandwich lunch.

The questionnaire was tested for face validity by inviting colleagues in the regional HTA center, Sahlgrenska University Hospital in Gothenburg, for comments. Two academically experienced senior consultants provided feedback in writing, and the questionnaire was discussed at an in-house HTA meeting. Furthermore, the questionnaire was tested by a general practitioner, who also provided feedback in writing.

### Statistical analysis

The statistical analyses were performed using SPSS (IBM SPSS Statistics for Windows, version 27.0, Armonk, NY). The internal consistency of items within Sections I, II, and V was analyzed using Cronbach’s alpha, with corrected item-total correlation noted for each item. Spearman’s correlation coefficient was calculated to explore the correlation between individual items in Section I and the corresponding items in Section II. We used the Mann–Whitney and chi-square tests for comparisons between groups. The results are presented as numbers (percentages) or medians (interquartile ranges). To facilitate interpretation, the mean ±  standard deviation was also provided where relevant, although normal distribution was not assumed in the statistical analyses. In the dichotomized analyses, students whose responses were 4 or 5 on the Likert scale were generally categorized as agreeing with the statement.

To investigate predictors of students’ perception that they had acquired adequate skills to incorporate scientific evidence into patient care, we performed logistic regression analyses to obtain crude and adjusted odds ratios (OR) with 95% confidence intervals (CI). The students’ dichotomized agreement with the statement “During the medical program, I have acquired sufficient skills in how to ground patient work on scientific evidence” was the dependent variable. We included the following individual characteristics as covariates in univariate and multivariate analyses: age (≤25 vs. > 25 years), gender (female vs. male), professional experience (yes vs. no), number of correct SBAs (≥4 vs. < 4) and having performed a systematic review for the master’s thesis (yes vs. no).

Concerning the students’ experience of the adequacy of the assessment regarding the 11 scholarly degree outcomes, we included variables in the multivariate analyses that statistically significantly predicted agreement with the statement in question in a multivariate model based solely on these outcomes. This approach was also applied to the educational content variables. Finally, we performed sensitivity analyses to investigate the robustness of the results. These analyses were based on a leave-one-out basis, excluding data from one university at a time. In additional sensitivity analyses, we omitted variables for which the OR shifted from > 1 in the crude analysis to < 1 in the main model or in any other sensitivity analysis. We also performed a sensitivity analysis in which the scholarly degree outcomes were omitted.

## Results

In total, 433 of the 641 students returned the questionnaire, resulting in a response rate of 68%. The student characteristics are presented in Table 1. Briefly, the median age was 25 years, and 255 (59%) students were female. The students had a median score of 3 correct answers on the SBAs (range 0‒5) (for details, see S1 Table).

**Table 1:** Questionnaire responses from 433 final-semester medical students.

				All	Agree sufficient skills^a^		
					Yes^b^ (n = 359)	No (n = 74)	P-value
Age				25 (24‒28) *(27 ± 4)*	25 (24‒28) *(27 ± 4)*	26 (24.5‒29) *(27 ± 4)*	0.12
Female sex				255 (59)	214 (60)	41 (59)	0.77
Worked as a junior physician				337 (78)	287 (82)	50 (69)	0.016
Master’s thesis was a systematic review				36 (8)	31 (9)	5 (7)	0.59
Single best answer questions (n = 5)		Number of correct answers		3 (2‒3) *(2.6 ± 1.1)*	3 (2‒3) *(2.6 ± 1.1)*	3 (2‒3) *(2.5 ± 1.0)*	0.50
		≥4 correct answers		83 (19)	72 (20)	11 (15)	0.30
Experience of adequacy of the assessment regarding scholarly degree outcomes during the medical program		Demonstrate knowledge of the scientific foundation of the field and insight into current research and development work as well as knowledge of the link between science and proven experience in professional practice	Response^a^	4 (3‒4) *(3.9 ± 0.9)*	4 (4‒5) *(4.0 ± 0.8)*	3 (3‒4) *(3.2 ± 1.0)*	<0.0001
			Agree^b^	303 (70)	279 (78)	24 (32)	<0.0001
		Demonstrate knowledge of fundamental scientific methodology in the field and insight into its opportunities and limitations	Response^a^	4 (4‒5) *(4.0 ± 0.9)*	4 (4‒5) *(4.1 ± 0.8)*	4 (3‒4) *(3.5 ± 1.0)*	<0.0001
			Agree^b^	323 (75)	283 (79)	40 (54)	<0.0001
		Demonstrate knowledge of ethical principles and their application in healthcare and research and development work	Response^a^	4 (4‒5) *(4.1 ± 0.9)*	4 (4‒5) *(4.2 ± 0.9)*	4 (3‒4) *(3.2 ± 1.0)*	0.0002
			Agree^b^	338 (78)	289 (81)	49 (66)	0.012
		Demonstrate knowledge of patient safety, quality, and prioritization in healthcare and methods for evaluating medical practice	Response^a^	4 (3‒5) *(3.8 ± 1.0)*	4 (3‒5) *(3.9 ± 0.9)*	4 (2.5‒4) *(3.3 ± 1.1)*	0.0001
			Agree^b^	290 (67)	253 (70)	37 (50)	0.001
		Demonstrate the ability to integrate and apply knowledge critically and systematically and analyze and assess complex phenomena, issues, and situations	Response^a^	4 (3‒5) *(3.9 ± 0.9)*	4 (4‒5) *(4.0 ± 0.8)*	4 (3‒4) *(3.5 ± 1.0)*	<0.0001
			Agree^b^	308 (71)	271 (75)	37 (50)	<0.0001
		Demonstrate the ability to initiate, participate in, and undertake improvement work as well as the necessary skills for participation in research and development work	Response^a^	4 (3‒4) *(3.5 ± 1.1)*	4 (3‒4) *(3.7 ± 1.0)*	3 (2‒4) *(2.8 ± 1.1)*	<0.0001
			Agree^b^	222 (51)	204 (57)	18 (24)	<0.0001
		Demonstrate advanced ability to discuss new data, phenomena, and issues in the field of medicine on a scientific basis with various audiences as well as critically review, assess, and utilize relevant information	Response^a^	4 (3‒5) *(3.9 ± 1.0)*	4 (3‒5) *(4.0 ± 0.9)*	3 (3‒4) *(3.4 ± 1.1)*	<0.0001
			Agree^b^	303 (70)	268 (75)	35 (47)	<0.0001
		Demonstrate the ability to use digital tools in both health care and research and development work	Response^a^	3 (2‒4) *(3.2 ± 1.2)*	3 (2‒4) *(3.3 ± 1.2)*	3 (2‒4) *(2.7 ± 1.2)*	0.0003
			Agree^b^	182 (42)	162 (45)	20 (27)	0.005
		Demonstrate the ability to self-reflect and empathize as well as have a professional attitude	Response^a^	5 (4‒5) *(4.3 ± 1.0)*	5 (4‒5) *(4.4 ± 0.9)*	4 (3‒5) *(3.9 ± 1.0)*	0.0001
			Agree^b^	361 (83)	308 (86)	53 (72)	0.005
		Demonstrate the ability to adopt a health-promoting approach with a holistic view of the patient based on a scientific perspective and with special consideration of ethical principles and human rights	Response^a^	4 (3‒5) *(4.0 ± 1.0)*	4 (4‒5) *(4.1 ± 0.9)*	4 (3‒4) *(3.6 ± 1.0)*	<0.0001
			Agree^b^	319 (74)	276 (77)	43 (58)	0.001
		Demonstrate the ability to identify the need for ongoing competence development and to take responsibility for it	Response^a^	4 (3‒5) *(3.9 ± 1.0)*	4 (3‒5) *(4.0 ± 0.9)*	3 (3‒4) *(3.4 ± 1.1)*	<0.0001
			Agree^b^	300 (69)	266 (74)	34 (46)	<0.0001
Educational content	Experience of having undergone education on HTA during the program		Response^a^	1 (1‒2) *(1.7 ± 1.0)*	1 (1‒2) *(1.8 ± 1.0)*	1 (1‒1) *(1.2 ± 0.5)*	<0.0001
			Agree^c^	87 (20)	85 (24)	2 (3)	<0.0001
	Experience of training during the medical program regarding components of a systematic review/HTA	To formulate a research question according to the PICO model	Response^a^	4 (2‒5) *(3.6 ± 1.4)*	4 (3‒5) *(3.7 ± 1.4)*	3 (2‒5) *(3.3 ± 1.5)*	0.040
			Agree^b^	250 (58)	215 (60)	35 (47)	0.041
		To find relevant literature according to the PICO model and after literature searches in relevant databases, such as PubMed and the Cochrane Library	Response^a^	4 (3‒5) *(3.7 ± 1.3)*	4 (3‒5) *(3.8 ± 1.3)*	3 (2‒4) *(3.2 ± 1.4)*	0.0001
			Agree^b^	271 (63)	238 (66)	33 (45)	0.0003
		To appraise scientific articles by using checklists	Response^a^	4 (3‒5) *(4.0 ± 1.0)*	4 (4‒5) *(4.1 ± 1.0)*	3.5 (3‒4) *(3.6 ± 1.1)*	<0.0001
			Agree^b^	308 (71)	272 (76)	36 (49)	<0.0001
		To synthesize results from multiple studies, e.g., in a meta-analysis	Response^a^	3 (2‒4) *(3.1 ± 1.4)*	3 (2‒4) *(3.2 ± 1.4)*	2 (1‒3) *(2.4 ± 1.2)*	<0.0001
			Agree^b^	172 (40)	156 (43)	16 (22)	0.001
		To assess evidence according to GRADE	Response^a^	3 (1‒4) *(2.8 ± 1.5)*	3 (2‒4) *(2.9 ± 1.5)*	2 (1‒3) *(2.1 ± 1.2)*	<0.0001
			Agree^b^	135 (31)	123 (34)	12 (16)	0.002
		To evaluate organizational aspects related to the implementation or withdrawal of a health technology in healthcare	Response^a^	3 (2‒4) *(2.9 ± 1.2)*	3 (2‒4) *(3.0 ± 1.2)*	2 (1‒3) *(2.3 ± 1.0)*	<0.0001
			Agree^b^	133 (31)	125 (35)	8 (11)	0.0001
		To evaluate economic aspects related to the implementation or withdrawal of a health technology in healthcare	Response^a^	3 (2‒3) *(2.7 ± 1.1)*	3 (2‒3) *(2.7 ± 1.1)*	2 (2‒3) *(2.5 ± 1.2)*	0.22
			Agree^b^	93 (21)	80 (22)	13 (18)	0.39
		To evaluate ethical aspects related to the implementation or withdrawal of a health technology in healthcare	Response^a^	4 (3‒5) *(3.8 ± 1.1)*	4 (3‒5) *(3.9 ± 1.1)*	4 (3‒4) *(3.5 ± 1.2)*	0.005
			Agree^b^	284 (66)	244 (68)	40 (54)	0.016
	Hands-on, credit-bearing, EBM-related learning activities integrated in clinical courses^d^			123 (28)	116 (32)	7 (9)	0.0001
	Bachelor’s and master’s theses, in all, 45 credits^e^ (as opposed to 30 credits for master’s thesis)			67 (15)	64 (18)	3 (4)	0.003

EBM =  evidence-based medicine; GRADE =  Grading of Recommendations, Assessment, Development, and Evaluations; HTA =  health technology assessment; SBA =  single best answer; PICO: P =  patients, I =  intervention, C =  comparison, O =  outcomes.

^a^From 1 = totally disagree to 5 = totally agree

^b^Responded 4 or 5 to the statement in question

^c^Responded 3‒5 to the statement “During the medical program, I underwent education on HTA” (requiring a 4‒5 response was not feasible, as all the responders agreed with the statement in question, i.e., “During the medical program, I have acquired sufficient skills in how to ground patient work on scientific evidence”)

^d^Two sites had such educational activities: Linköping University, Lund University.

^e^Two sites: Lund University, Örebro University

Overall and according to their agreement with the statement “During the medical program, I have acquired sufficient skills in how to ground patient work on scientific evidence.” The results are presented as numbers (percentages) or medians (interquartile ranges). To facilitate interpretation, the mean ±  standard deviation is presented in italics in parentheses where applicable.

### Experience regarding degree outcomes and training

Overall, many students experienced that they had been adequately assessed regarding the scholarly degree outcomes (Table 1). For instance, 361 (83%) students reported adequate assessment of the outcome related to self-reflection and professional attitude, and the corresponding proportion for eight other outcomes ranged between 67% and 78%. By contrast, half or less than half of the students reported having been adequately assessed in the area of skills related to two scholarly degree outcomes: improvement work and the use of digital tools. Further, the comprehensibility of the degree outcomes was generally good (see S2 Table). All corresponding items in Sections I and II, except for the item regarding the use of digital tools, were correlated (correlation coefficients: 0.30‒0.35, all P < 0.0001; see S3 Table).

Concerning learning activities in the procedural steps of a systematic review, 250 (58%) students stated that they had been trained to define a specific research question using the PICO (P =  patients, I =  intervention, C =  comparison, O =  outcomes) format; 271 (63%) stated that they had been trained to find relevant literature pertinent to a specific research question; 308 (71%) stated that they had been trained to appraise scientific articles using checklists; 172 (40%) stated that they had been trained to synthesize results from multiple studies; and 135 (31%) stated that they had been trained to assess the certainty of evidence according to GRADE (Table 1). By contrast, only 26 (6%) students reported having been trained in the area of HTA, and 87 (20%) students provided a rating at a level of agreement of 3 or higher on this statement. Regarding training in HTA-specific aspects not included in a systematic review, 133 (31%), 93 (21%), and 284 (66%) students reported having been trained in the evaluation of organizational, economic, and ethical aspects, respectively, related to the introduction or withdrawal of health technologies in healthcare.

For items in Sections I, II, and V, the Cronbach’s alpha ranged between 0.81 and 0.86 (see S4 Table). The corrected item‒total correlation ranged between 0.41 and 0.63 (Section I), 0.40 and 0.62 (Section II), and 0.43 and 0.62 (Section V).

### Perceived skills in EBM and HTA

Overall, 359 (83%) students perceived that they had developed sufficient skills in how to ground patient work on scientific evidence. In the univariate model, the students’ experience of having been adequately assessed on all scholarly degree outcomes predicted agreement with the statement “During the medical program, I have acquired sufficient skills in how to ground patient work on scientific evidence” (Table 2). However, when the individual items were included in the same model, the statistical significance remained for only two outcomes (see S4 Table). Correspondingly, almost all items concerning the students’ experience of scientifically related educational content predicted agreement with their perception of having been sufficiently prepared to incorporate scientific evidence into patient care. Nevertheless, when these items were included in the same model, the statistical significance remained constant for only four of them (see S5 Table).

**Table 2:** Predictors of final-semester students agreeing with the statement “During the medical program, I have acquired sufficient skills in how to ground patient work on scientific evidence.”.

			OR (95% CI)	aOR^a^ (95% CI)
Individual characteristics	Age (≤25 vs. > 25 years)		1.51 (0.90; 2.55)	1.51 (0.76; 3.03)
	Sex (female vs. male)		1.08 (0.64; 0.82)	1.13 (0.58; 2.22)
	Worked as a junior physician (yes vs. no)		**2.00 (1.13; 3.55)**	1.61 (0.74; 3.50)
	Single best answer questions (≥4 vs. < 4 correct answers)		1.08 (0.85; 1.37)	1.14 (0.45; 2.87)
	Master’s thesis was a systematic review (yes vs. no)		1.31 (0.49; 3.48)	0.98 (0.25; 3.92)
Experience of the adequacy of the assessment regarding scholarly degree outcomes during the medical program^b^	Demonstrate knowledge of the scientific foundation of the field and insight into current research and development work as well as knowledge of the link between science and proven experience in professional practice		**7.60 (4.38; 13.2)**	**6.17 (3.10; 12.3)**
	Demonstrate knowledge of fundamental scientific methodology in the field and insight into its opportunities and limitations		**3.20 (1.89; 5.42)**	
	Demonstrate knowledge of ethical principles and their application in health care and research and development work		**2.02 (1.16; 3.52)**	
	Demonstrate knowledge of patient safety, quality, and prioritization in health care, and methods for evaluating medical practice		**2.34 (1.40; 3.91)**	
	Demonstrate the ability to integrate and apply knowledge critically and systematically and analyze and assess complex phenomena, issues, and situations		**3.14 (1.87; 5.28)**	
	Demonstrate the ability to initiate, participate in, and undertake improvement work as well as the necessary skills for participation in research and development work		**3.85 (2.17; 6.85)**	1.76 (0.86; 3.59)
	Demonstrate advanced ability to discuss new data, phenomena, and issues in the field of medicine on a scientific basis with various audiences as well as critically review, assess, and utilize relevant information		**3.28 (1.96; 5.49)**	
	Demonstrate the ability to use digital tools in both health care and research and development work		**2.21 (1.26; 3.86)**	
	Demonstrate the ability to self-reflect and empathize as well as have a professional attitude		**2.62 (1.28; 4.21)**	
	Demonstrate the ability to adopt a health-promoting approach with a holistic view of the patient based on a scientific perspective and with special consideration of ethical principles and human rights		**2.35 (1.39; 3.98)**	
	Demonstrate the ability to identify the need for ongoing competence development and to take responsibility for it		**3.34 (1.98; 5.63)**	
Educational content	Experience having undergone education on HTA during the program^c^		**11.2 (2.68; 46.5)**	**11.3 (1.44; 89.5)**
	Experience of having been trained during the medical program regarding the component in question^b^	To formulate a research question according to the PICO model	**1.70 (1.02; 2.84)**	
		To find relevant literature according to the PICO model and after literature searches in relevant databases, such as PubMed and the Cochrane Library	**2.55 (1.53; 4.26)**	
		To appraise scientific articles by using checklists	**3.16 (1.88; 5.33)**	**2.46 (1.23; 4.90)**
		To synthesize results from several studies, e.g., in a meta-analysis	**2.69 (1.49; 4.88)**	
		To assess evidence according to GRADE	**2.76 (1.42; 5.37)**	
		To assess organizational aspects related to the introduction or withdrawal of a health technology in healthcare	**4.41 (2.04; 9.54)**	**2.65 (1.05; 6.67)**
		To assess economic aspects related to the introduction or withdrawal of a health technology in healthcare	1.33 (0.69; 2.54)	
		To assess ethical aspects related to the introduction or withdrawal of a health technology in healthcare	**1.89 (1.12; 3.19)**	
	Hands-on, credit-bearing, EBM-related learning activities integrated in clinical courses (yes vs. no)		**4.57 (2.03; 10.3)**	**4.68 (1.69; 13.0)**
	Bachelor and master theses (yes vs. no)		**5.13 (1.57; 16.8)**	

aOR =  adjusted odds ratio; EBM =  evidence-based medicine; GRADE =  Grading of Recommendations, Assessment, Development, and Evaluations; HTA =  health technology assessment; OR =  odds ratio (crude); PICO: P =  patients, I =  intervention, C =  comparison, O =  outcomes.

^a^The multivariate regression model included (i) all individual characteristic variables; (ii) scholarly degree outcome assessments that statistically significantly predicted agreement with the statement “During the medical program, I developed sufficient skills in applying scientific evidence in patient care” in a logistic regression model with these variables only (S1 Table), and (iii) educational content variables that statistically significantly predicted agreement with the same statement in a logistic regression model with these variables only (S1 Table).

^b^Agreed (vs. disagreed) to having been adequately assessed on the degree outcome in questions or having undergone training regarding the component in question during the medical program: defined as responding 4 or 5 (vs. 1‒3) on a scale from 1 =  totally disagree to 5 =  totally agree.

^c^Agreed (vs. disagreed) to having education on HTA: defined as responding 3‒5 (vs. 1‒2) on a scale from 1 =  totally disagree to 5 =  totally agree (requiring a 4‒5 response was not feasible, as all the responders agreed with the statement in question, i.e., “During the medical program, I have acquired sufficient skills in how to ground patient work on scientific evidence”)

In the multivariate model, the students’ experience of the adequacy of the assessment regarding the scholarly degree outcome (i.e., “Demonstrate knowledge about the scientific foundation of medicine, insights into current research as well as knowledge of the link between science and proven experience”) predicted their perception of having developed sufficient skills in incorporating scientific evidence into patient care (Table 2). Additional predictors included the experience of having undergone education on HTA during the program as well as training in scientific skills related to two procedural steps of a systematic review/HTA, that is, appraising scientific articles by using checklists and assessing organizational aspects associated with the implementation or withdrawal of health technologies in healthcare. Finally, the presence of hands-on, credit-bearing, EBM-related learning activities during clinical courses was also a predictor. The sensitivity analyses showed that the results were robust (see S6 Table).

## Discussion

This cross-sectional study of six medical programs in Sweden, comprising more than 400 final-semester medical students, shows that most students experienced having been adequately assessed on scientifically related degree outcomes. Reported adequate assessment on the scholarly outcome “Demonstrate knowledge about the scientific foundation of medicine…” predicted students’ perception of having developed sufficient skills in incorporating scientific evidence into patient care. Additional predictors included learning activities related to systematic reviews and HTAs as well as hands-on, credit-bearing, EBM-related learning activities during clinical courses. Surprisingly few students experienced that they had been trained in the area of HTA, and a non-negligible proportion reported not having been trained in the important procedural steps of a systematic review.

In medical programs, assessment of scholarly degree outcomes is critical as scientific principles underpin virtually every aspect of medical practice, guiding physicians, for instance, during the process of differential diagnosis and in decision-making regarding treatment alternatives and follow-up strategies. In addition, assessment per se is an essential part of constructive alignment, meaning that learning outcomes, teaching activities, and assessments are aligned with each other [17]. Our finding that the assessment of the scholarly degree outcome (i.e., “Demonstrate knowledge about the scientific foundation of medicine…”) was key to students’ perception of having acquired sufficient skills in incorporating scientific evidence into patient care underscores the crucial role of well-thought-out assessments. Although the importance of this degree outcome may not be surprising, given the inclusion of the broad scope of “the scientific foundation of medicine”, the results can provide valuable insights for constructive alignment.

The findings of this study suggest that hands-on EBM-related learning activities during clinical courses, including assessments that contribute credits toward graduation, have significant educational value. Again, this is in line with constructive alignment. Furthermore, contextual learning acknowledges the importance of embedding learning into an authentic setting, thereby making it more meaningful to students in their future profession [18,19]. Indeed, hands-on, credit-bearing, EBM-related learning activities during clinical courses predicted students’ perceptions of having acquired sufficient skills in applying scientific evidence in patient care. This suggests that integrating EBM teaching into clinical courses may help students understand how scientific findings are used in clinical practice. The benefits of hands-on EBM-related learning activities are also in line with the findings of systematic review overviews concluding that EBM learning strategies should be multifaceted and integrated into the clinical context [20,21]. More recent studies also show benefits of learning activities of EBM both early and late in the medical program [22–24]. Moreover, our finding that repeated learning activities across multiple clinical courses can enhance students’ learning of EBM aligns with recent research indicating that EBM habits and knowledge tend to decline after a single instructional program in the 4th or 5th year of the medical program [24].

A graduate physician in modern health care is typically exposed to numerous guidelines, which should be based on a systematic evidence synthesis [25]. Given that the rigor of development, including the approach to evidence synthesis, is a key domain in assessing the quality of a guideline [26] and, consequently, its applicability for a physician as part of EBM, it may be surprising that a non-negligible proportion of the final-semester medical students reported that they had not been trained in the steps required to perform a systematic review. For instance, 70% reported not having been trained to assess evidence using the GRADE system. In this context, it may be of interest to note that the self-perceived competence of medical students and physicians was often low in the EBM domains of analysis and application [27]. Given that GRADE is recommended for determining the certainty of evidence [28], and that interpreting certainty and strength of recommendations are key competencies within evidence-based practice, our findings suggest that this area of education may need greater emphasis in curriculum development [29]. The fact that many guidelines have apparent quality problems regarding the underlying evidence synthesis [30] further underscores the need to include training within the procedural steps of systematic reviews in the curriculum.

Notably, 60% of the medical students indicated that they had not been trained to synthesize results from multiple studies, for example, in a meta-analysis. In the absence of experience, graduate physicians may find it difficult to distinguish useful meta-analyses from flawed ones. Arguably, when this educational content is lacking, EBM in clinical practice may be hampered, especially as only 3% of published meta-analyses are reportedly decent and clinically useful [31]. Again, identifying and critically appraising key elements of systematic reviews and interpreting presentations of pooled results have been described as core competencies of evidence-based practice [29]. Furthermore, these aspects are considered to require practical training during the curriculum [29], illustrating the challenges involved.

Regarding the appraisal of scientific articles using checklists, our findings are more encouraging. About 70% of the medical students reported having received training within this component of a systematic review/HTA. This educational content therefore seems to have been largely implemented in medical programs. However, since these results reflect a core competency—the ability to critically evaluate the integrity, reliability, and applicability of health-related research [29]—the reverse finding, that 30% of the students reported not to have been trained implies that there is room for curriculum improvement.

Although HTA is not currently described as a core competency for health professionals, it is intricately linked to EBM. From a medical student perspective, knowledge about HTA may contribute insights to future professional life beyond evidence synthesis. Indeed, an HTA often also includes economic, societal, and ethical aspects and informs healthcare decision-making at the organizational level. Undoubtedly, such decisions have implications for physicians’ everyday practice. In the context of medical programs, a deep understanding of other academic disciplines, sucjh as health economics, is not required. However, an understanding of overarching prioritization principles is essential. Our finding that many final-semester medical students interpreted the concept of cost-effectiveness erroneously may therefore suggest that HTA and prioritization principles deserve increased attention in learning activities during medical school.

A notable strength of this study is that it contributes knowledge regarding predictors of the perception of having developed adequate skills to incorporate scientific evidence into patient care, a core aspect of EBM. Furthermore, the study achieved a relatively high response rate from students across six medical programs. Thus, the findings can be acceptably generalizable. Nevertheless, it must be noted that students who chose not to participate in this study could have reported worse perceptions than those who did. In addition, they could have performed worse on the SBAs. Indeed, an original study with an 88% participation rate, including medical as well as other students, reported that 64% of those participating passed the subsequent exam whereas only 15% of those not participating did so [32]. Nevertheless, the sensitivity analysis—in which the site with a considerably low response rate was excluded—yielded similar results. Furthermore, our results were robust in the other sensitivity analyses. Finally, the results related to internal consistency suggest that the scales used in the questionnaire were reliable.

An important limitation of the present study is that our results reflect students’ perceptions of having developed sufficient skills in incorporating scientific evidence into patient care and not the actual assessment of these skills. Indeed, self-reported data comprising students’ perceptions and experiences are inherently subjective and possibly influenced by factors such as emotions and context, and there is an obvious risk of recall bias as the medical program spans several years. For future evaluations, using the Fresno test could provide valuable insights into students’ knowledge and skills in EBM as well as the impact of teaching interventions [33]. Other limitations include the cross-sectional design, which allows conclusions regarding associations but not causality. However, self-reported data are regularly used in research and can allow for quantitative analyses. Furthermore, it should be noted that we did not comprehensively map or analyze the assessment methods applied in the scholarly degree outcomes at the universities under study. Indeed, the study was limited to the questionnaire data and the overall mapping of the included programs; therefore, the possibility that other factors may also be of importance cannot be excluded. Finally, a potential limitation was that questions regarding the awareness and knowledge of regulatory assessment documentation as a source of scientific evidence were not included in the questionnaire. This may be particularly relevant given the new HTA regulation, which indicates closer collaboration and the use of convergent methodologies [34]. Additionally, the establishment of the Data Analysis and Real World Interrogation Network within the European Union (DARWIN EU) highlights a focus on leveraging evidence from healthcare databases across the EU [35]. Nevertheless, the SBAs addressing the interpretation of a meta-analysis using GRADE to evaluate the certainty of evidence, a forest plot with a meta-analysis, and a health economics statement on cost-effectiveness may partially capture the ability of final-year medical students to interpret regulatory assessment documentation. Furthermore, the SBA regarding the interpretation of a case/control study based on register data may provide some information related to DARWIN EU.

## Conclusions

Scientific principles form the foundation of nearly every aspect of medical practice. Our findings suggest that there should be an increased focus on educational efforts in medical programs aimed at supporting the learning process of medical students in perceiving themselves as prepared to incorporate scientific evidence into patient care: (i) ensuring assessment of key content relating to scholarly outcomes, including the scientific foundation of medicine; (ii) incorporating learning activities on HTA and the systematic review process; and (iii) integrating hands-on, credit-bearing, EBM-related learning activities into clinical courses.

## Supporting information

S1 File Questionnaire. (DOCX)

S1 Table Results SBA. (DOCX)

S2 Table Comprehensibility of scholarly degree outcomes. (DOCX)

S3 Table Correlations. (DOCX)

S4 Table Internal consistency. (DOCX)

S5 Table Regression analyses of variables for the final model. (DOCX)

S6 Table Sensitivity analyses. (DOCX)

S1 Data Details about questionnaire data used in the analyses. (DOCX)
