# Supplementary material for: Preparing medical students to incorporate scientific evidence into patient care: A cross-sectional study
Source: PLoS One. 2025 Apr 4;20(4):e0321211. doi: 10.1371/journal.pone.0321211 (PMC11970701; doi:10.1371/journal.pone.0321211)
Supplement: S1 File — (DOCX) [file pone.0321211.s001.docx]

**Questionnaire to students at the end of medical programs in Sweden**

| **Mark (by ticking one box per degree outcome) to what extent you agree with the following statements**  *(on a scale from ”Totally agree” to ”Totally disagree”)*  **1.** I experience that I have been adequately **assessed** during the medical program regarding to... | *5. Totally agree* | |  | |  | *1. Totally disagree* | | *Do not know* |
| --- | --- | --- | --- | --- | --- | --- | --- | --- |
| **a.** ... demonstrate knowledge of the scientific foundation of the field and insight into current research and development work as well as knowledge of the link between science and proven experience in professional practice | □ | □ | | □ | □ | | □ | □ |
| **b.** ... demonstrate knowledge of fundamental scientific methodology in the field and insight into its opportunities and limitations | □ | □ | | □ | □ | | □ | □ |
| **c.** ... demonstrate knowledge of ethical principles and their application in healthcare and research and development work | □ | □ | | □ | □ | | □ | □ |
| **d.** ... demonstrate knowledge of patient safety, quality, and prioritization in healthcare and methods for evaluating medical practice | □ | □ | | □ | □ | | □ | □ |
| **e.** ... demonstrate the ability to integrate and apply knowledge critically and systematically and analyze and assess complex phenomena, issues, and situations | □ | □ | | □ | □ | | □ | □ |
| **f.** ... demonstrate the ability to initiate, participate in, and undertake improvement work as well as the necessary skills for participation in research and development work | □ | □ | | □ | □ | | □ | □ |
| **g.** ... demonstrate advanced ability to discuss new data, phenomena, and issues in the field of medicine on a scientific basis with various audiences as well as critically review, assess, and utilize relevant information | □ | □ | | □ | □ | | □ | □ |
| **h.** ... demonstrate the ability to use digital tools in both health care and research and development work | □ | □ | | □ | □ | | □ | □ |
| **i.** ... demonstrate the ability to self-reflect and empathize as well as have a professional attitude | □ | □ | | □ | □ | | □ | □ |
| **j.** ... demonstrate the ability to adopt a health-promoting approach with a holistic view of the patient based on a scientific perspective and with special consideration of ethical principles and human rights | □ | □ | | □ | □ | | □ | □ |
| **k.** … demonstrate the ability to identify the need for ongoing competence development and to take responsibility for it | □ | □ | | □ | □ | | □ | □ |

| **Mark (by ticking one box per degree outcome) to what extent you understand the content**  *(on a scale from ”Very easy to understand” to ”Very hard to understand”)*  **2.** What do you think of the **wordings** of the following scholarly degree objectives: | *5.*  *Very easy to understand* | |  | |  | *1.*  *Very hard to understand* | |
| --- | --- | --- | --- | --- | --- | --- | --- |
| **a.** To demonstrate knowledge of the scientific foundation of the field and insight into current research and development work as well as knowledge of the link between science and proven experience in professional practice | □ | □ | | □ | □ | | □ |
| **b.** To demonstrate knowledge of fundamental scientific methodology in the field and insight into its opportunities and limitations | □ | □ | | □ | □ | | □ |
| **c.** To demonstrate knowledge of ethical principles and their application in healthcare and research and development work | □ | □ | | □ | □ | | □ |
| **d.** To demonstrate knowledge of patient safety, quality, and prioritization in healthcare and methods for evaluating medical practice | □ | □ | | □ | □ | | □ |
| **e.** To demonstrate the ability to integrate and apply knowledge critically and systematically and analyze and assess complex phenomena, issues, and situations | □ | □ | | □ | □ | | □ |
| **f.** To demonstrate the ability to initiate, participate in, and undertake improvement work as well as the necessary skills for participation in research and development work | □ | □ | | □ | □ | | □ |
| **g.** To demonstrate advanced ability to discuss new data, phenomena, and issues in the field of medicine on a scientific basis with various audiences as well as critically review, assess, and utilize relevant information | □ | □ | | □ | □ | | □ |
| **h.** To demonstrate the ability to use digital tools in both health care and research and development work | □ | □ | | □ | □ | | □ |
| **i.** To demonstrate the ability to self-reflect and empathize as well as have a professional attitude | □ | □ | | □ | □ | | □ |
| **j.** To demonstrate the ability to adopt a health-promoting approach with a holistic view of the patient based on a scientific perspective and with special consideration of ethical principles and human rights | □ | □ | | □ | □ | | □ |
| **k.** To demonstrate the ability to identify the need for ongoing competence development and to take responsibility for it | □ | □ | | □ | □ | | □ |

| **Mark (by ticking one box per row) to what extent you agree with the following statements regarding professional work as a physician according to science and proven experience:**  *(on a scale from ”Totally agree” to ”Totally disagree”)* | *5. Totally agree* | |  | |  | *1. Totally disagree* | |
| --- | --- | --- | --- | --- | --- | --- | --- |
| **3.** During the medical program, I have acquired sufficient skills in how to ground patient work on scientific evidence | □ | □ | | □ | □ | | □ |
| **4.** During the medical program, I have undergone education on HTA (health technology assessment) | □ | □ | | □ | □ | | □ |

| **5.** **Mark (by ticking one box per row) to what extent you agree with the following statements regarding the performance of a systematic review:**  *(on a scale from ”Totally agree” to ”Totally disagree”. Do NOT include the mandatory research project(s))* |  | | |  | |  |  | |  |
| --- | --- | --- | --- | --- | --- | --- | --- | --- | --- |
| During the medical program, I have been **trained**... | | *5. Totally agree* | |  | |  | *1. Totally disagree* | | *Do not know* |
| **a.** ... to formulate a research question according to PICO (P=patients, I=intervention, C=comparison, O=outcomes) model | □ | | □ | | □ | □ | | □ | □ |
| **b.** ... to find relevant literature according to the PICO model and after literature searches in relevant databases, such as PubMed and the Cochrane Library | □ | | □ | | □ | □ | | □ | □ |
| **c.** ... to appraise scientific articles by using checklists | □ | | □ | | □ | □ | | □ | □ |
| **d.** ... to synthesize results from multiple studies, e.g., in a meta-analysis | □ | | □ | | □ | □ | | □ | □ |
| **e.** ... to assess evidence according to GRADE | □ | | □ | | □ | □ | | □ | □ |

| **6.** **Mark (by ticking one box per row) to what extent you agree with the following statements regarding the implementation/withdrawal of a health technology in healthcare (diagnostics/treatment):**  *(on a scale from ”Totally agree” to ”Totally disagree”. Do NOT include the mandatory research project(s))* | | |  | |  | |  |  | |  |
| --- | --- | --- | --- | --- | --- | --- | --- | --- | --- | --- |
| During the medical program, I have been **trained**... | | *5. Totally agree* | | |  | |  | *1. Totally disagree* | | *Do not know* |
| **a.** ... to evaluate organizational aspects related to the implementation or withdrawal of a health technology in healthcare (treatment/diagnostics) | □ | | | □ | | □ | □ | | □ | □ |
| **b.** ...to evaluate economic aspects related to the implementation or withdrawal of a health technology in healthcare (treatment/diagnostics) | □ | | | □ | | □ | □ | | □ | □ |
| **c.** .. to evaluate ethical aspects related to the implementation or withdrawal of a health technology in healthcare (treatment/diagnostics) | □ | | | □ | | □ | □ | | □ | □ |

**Now five questions will follow, related to the scientific basis of the profession as a physician**

**7.** You are reading a scientific study where data were retrieved from several registers and linked using the personal identity number. Among 1,000 patients who had had a myocardial infarction, 50 had used liraglutide during the three months preceding the myocardial infarction, and in 1,000 randomly selected patients who had not had a myocardial infarction, the corresponding number was 10, i.e. 10 had used liraglutide. The patients with and without myocardial infarction were of similar age and the distribution between women and men was similar in both groups.

Which option is most reasonable to describe this study?

| □ | This non-randomized controlled trial shows that there is an association between myocardial infarction and liraglutide |
| --- | --- |
| □ | This matched cohort study shows that the risk of myocardial infarction is five times greater if treated with liraglutide |
| □ | This case-control study shows that withdrawal of liraglutide reduces the risk of myocardial infarction |
| □ | This randomized controlled trial shows that treatment with liraglutide increases the risk of myocardial infarction |
| □ | The design of this study is particularly appropriate for investigating causal relationships |

**8.** Your patient is concerned about a new blood disease that has been found in 10% of the population. He is now wondering if there is any good method to determine if he has it. You read about a new diagnostic method for early detection of this disease and find a blood test that costs SEK 15,000. An evidence synthesis shows that the sensitivity is 0.78 and the specificity 0.44. How do you describe the results to your patient?

Which option is most reasonable?

| □ | Even if this test turns out positive, there is an obvious risk that you do not have the disease, that is, that the test shows a false positive result |
| --- | --- |
| □ | This test is good at differentiating between those who have the disease and those who do not |
| □ | This test captures about half of those who have the disease |
| □ | In healthcare, such an expensive diagnostic method is never used; you will have to wait for the company to request a lower price |
| □ | The positive predictive value of the current test is likely to be high |

**9.** Your patient is scheduled for hip replacement surgery due to osteoarthritis after many years of elite level badminton training. She has read in the newspaper that a new surgical method, although significantly more expensive, results in fewer complications, and is wondering about your opinion on this. You find the systematic review behind the newspaper headline. It reports the pooled results of three randomized trials showing that the risk ratio for complications for the new surgical method, compared with the old one, is 0.54 (95% confidence interval: 0.29 to 0.98). The risk difference is

-0.0041 (-0.0082 to -0.00001), or, expressed in percentage points: -0.4 (-0.8 to -0.001). The certainty of evidence is reported according to the GRADE system (Grading of Recommendations, Assessment, Development and Evaluations) as ⊕⊕ΟΟ. How would you describe the results to your patient?

Which option is most reasonable?

| □ | Overall, the studies that have been done show that the risk of complications is similar with the new method as with the old one |
| --- | --- |
| □ | Overall, the studies that have been done show that it is possible that slightly fewer patients will have complications with the new method, but the absolute difference is generally small |
| □ | Overall, the studies that have been done show that the new method probably involves significant risks; almost twice as many patients can be expected to experience complications |
| □ | Overall, the studies that have been done show, with high certainty of evidence, that the risks are significantly lower with the new method |
| □ | It is not possible to draw any conclusions about the risk of complications based on the studies that have been carried out |

**10.** Two forest plots are presented below, from two types of meta-analyses of six randomized controlled trials comparing clopidogrel with ticagrelor after acute coronary syndrome with regard to the risk of a clinically significant bleeding.

A: Risk ratio

**
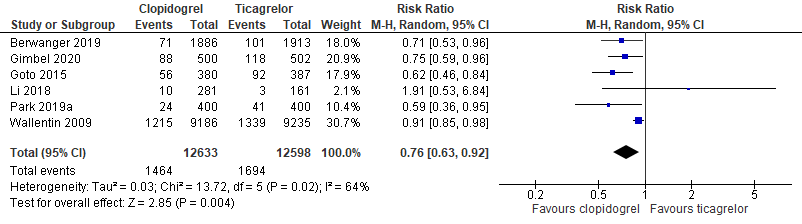
**

B: Risk difference

**
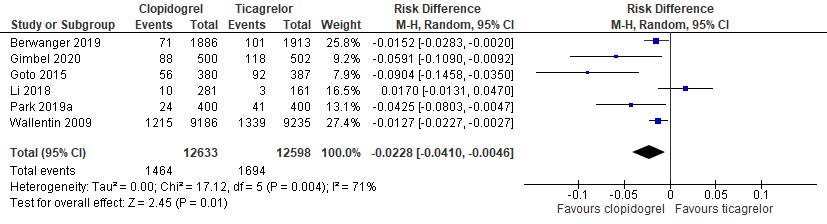
**

Which option is the most reasonable interpretation of these plots?

| □ | All studies included in the meta-analyses show the same results; statistically significant lower risk of a clinically significant bleeding with clopidogrel compared to ticagrelor |
| --- | --- |
| □ | The meta-analysis shows that, relatively speaking, there is a 2.3 percent lower risk of clinically significant bleeding with clopidogrel compared to ticagrelor |
| □ | On average, close to 25 patients need to be treated with clopidogrel instead of ticagrelor to avoid one (1) clinically significant bleeding |
| □ | The study “Gimbel 2020” contributes more to the meta-analysis regarding absolute risk difference than to the meta-analysis regarding relative effects |
| □ | With 95% probability, treatment with clopidogrel instead of ticagrelor reduces the absolute risk of a clinically significant bleeding by 0.5 to 4.1 percentage points |

**11.** You read on the Internet that a new drug against lung cancer will be included in the drug reimbursement system because it has been judged to be cost-effective, with a cost per quality-adjusted life year of SEK 1 million.

Which option is the most reasonable consequence for healthcare?

| □ | If this drug is used, it may mean that available resources for other things may decrease |
| --- | --- |
| □ | This drug should be used because the National Board of Health and Welfare has decided that it is included in the guidelines |
| □ | The drug should be used for all patients because it has been shown to improve survival |
| □ | The price of the drug can be expected to increase by time as the pharmaceutical company has set a low starting price |
| □ | The new drug implies a cost saving for healthcare |

**At last, some questions about your background**

**12.** The following components during the medical program have helped me to develop skills to work, as a physician, according to science and proven experience. (Answer in free text)

__________________________________________________________________________________

__________________________________________________________________________________

__________________________________________________________________________________

__________________________________________________________________________________

__________________________________________________________________________________

__________________________________________________________________________________

__________________________________________________________________________________

__________________________________________________________________________________

**13.** To better prepare myself as a physician in clinical practice to work according to science and proven experience, it would be of helpful if... (Answer in free text)

__________________________________________________________________________________

__________________________________________________________________________________

__________________________________________________________________________________

__________________________________________________________________________________

__________________________________________________________________________________

__________________________________________________________________________________

__________________________________________________________________________________

__________________________________________________________________________________

**14.** I am □ a woman

□ a man

□ other/no answer

**15.** I am _____ years old

**16.** I **□** do not have a doctoral degree

**□** have a doctoral degree

**17.** I have studied the entire medical program at the same university

**□** yes

**□** no, I have also studied at________________________________________________

*(state university where you studied medicine, except the present one)*

**18.** My mandatory research project was a systematic review

□ yes

□ no

**19.** I have worked for____________________ months as an assistant physician

*(state a number)*

*Thank you for your participation!*
