# Supplementary material for: Preparing medical students to incorporate scientific evidence into patient care: A cross-sectional study
Source: PLoS One. 2025 Apr 4;20(4):e0321211. doi: 10.1371/journal.pone.0321211 (PMC11970701; doi:10.1371/journal.pone.0321211)
Supplement: S1 Table — (DOCX) [file pone.0321211.s002.docx]

**S1 Table.** Number (percentage) of the students that responded correctly to the five single-best-answer (SBA) questions.

| **Regarding the interpretation of…** | **n (%)** |
| --- | --- |
| …a case/control study | 118 (27) |
| …a diagnostic study | 361 (83) |
| …a meta-analysis with certainty of evidence expressed according to GRADE | 311 (72) |
| …a forest plot including a meta-analysis | 94 (22) |
| …a health economics statement regarding cost-effectiveness | 222 (51) |

GRADE = Grading of Recommendations, Assessment, Development, and Evaluations
