# Supplementary material for: Preparing medical students to incorporate scientific evidence into patient care: A cross-sectional study
Source: PLoS One. 2025 Apr 4;20(4):e0321211. doi: 10.1371/journal.pone.0321211 (PMC11970701; doi:10.1371/journal.pone.0321211)
Supplement: S2 Table — (DOCX) [file pone.0321211.s003.docx]

**S2 Table.** Questionnaire responses from 433 final-semester medical students regarding their agreement with the statement “What do you think of the wordings of the following scholarly degree objectives”, with the endpoints 1 = “very hard to understand” and 5 = “very easy to understand”. The results are presented as numbers (percentages) or medians (interquartile ranges). To facilitate interpretation, the mean ± standard deviation is presented in italics.

| Demonstrate knowledge of the scientific foundation of the field and insight into current research and development work as well as knowledge of the link between science and proven experience in professional practice | Response | 4 (3‒4)  *(3.5 ± 1.0)* |
| --- | --- | --- |
|  | Agree^a^ | 219 (51) |
| Demonstrate knowledge of fundamental scientific methodology in the field and insight into its opportunities and limitations | Response | 4 (4‒5)  *(4.1 ± 0.9)* |
|  | Agree^a^ | 330 (76) |
| Demonstrate knowledge of ethical principles and their application in healthcare and research and development work | Response | 5 (4‒5)  *(4.3 ± 0.8)* |
|  | Agree^a^ | 366 (85) |
| Demonstrate knowledge of patient safety, quality, and prioritization in healthcare and methods for evaluating medical practice | Response | 4 (4‒5)  *(4.1 ±.09)* |
|  | Agree^a^ | 335 (77) |
| Demonstrate the ability to integrate and apply knowledge critically and systematically and analyze and assess complex phenomena, issues, and situations | Response | 4 (3‒4)  *(3.5 ± 1.1)* |
|  | Agree^a^ | 234 (54) |
| Demonstrate the ability to initiate, participate in, and undertake improvement work as well as the necessary skills for participation in research and development work | Response | 4 (3‒4)  *(3.7 ± 1.1)* |
|  | Agree^a^ | 256 (59) |
| Demonstrate advanced ability to discuss new data, phenomena, and issues in the field of medicine on a scientific basis with various audiences as well as critically review, assess, and utilize relevant information | Response | 4 (3‒5)  *(3.9 ± 1.0)* |
|  | Agree^a^ | 310 (72) |
| Demonstrate the ability to use digital tools in both health care and research and development work | Response | 4 (3‒5)  *(3.9 ± 1.1)* |
|  | Agree^a^ | 298 (69) |
| Demonstrate the ability to self-reflect and empathize as well as have a professional attitude | Response | 5 (4‒5)  *(4.5 ± 0.8)* |
|  | Agree^a^ | 376 (87) |
| Demonstrate the ability to adopt a health-promoting approach with a holistic view of the patient based on a scientific perspective and with special consideration of ethical principles and human rights | Response | 4 (3‒5)  *(4.0 ± 1.0)* |
|  | Agree^a^ | 312 (72) |
| Demonstrate the ability to identify the need for ongoing competence development and to take responsibility for it | Response | 5 (4‒5)  *(4.2 ± 0.9)* |
|  | Agree^a^ | 347 (80) |

^a^Responded 4 or 5 to the statement in question
