# Supplementary material for: Preparing medical students to incorporate scientific evidence into patient care: A cross-sectional study
Source: PLoS One. 2025 Apr 4;20(4):e0321211. doi: 10.1371/journal.pone.0321211 (PMC11970701; doi:10.1371/journal.pone.0321211)
Supplement: S4 Table — (DOCX) [file pone.0321211.s005.docx]

**S4 Table.** Internal consistency for items regarding the experience of adequate assessment and comprehensibility of scholarly degree outcomes as well as training of components of a systematic review/HTA.

|  |  | **Corrected item-total correlation** | **Crohnbach’s alpha** |
| --- | --- | --- | --- |
| Experience of the adequacy of the assessment regarding scholarly degree outcomes during the medical program^a^ (n=401) | Demonstrate knowledge of the scientific foundation of the field and insight into current research and development work as well as knowledge of the link between science and proven experience in professional practice | 0.56 | 0.86 |
|  | Demonstrate knowledge of fundamental scientific methodology in the field and insight into its opportunities and limitations | 0.53 |  |
|  | Demonstrate knowledge of ethical principles and their application in health care and research and development work | 0.63 |  |
|  | Demonstrate knowledge of patient safety, quality, and prioritization in healthcare and methods for evaluating medical practice | 0.63 |  |
|  | Demonstrate the ability to integrate and apply knowledge critically and systematically, and analyze and assess complex phenomena, issues, and situations | 0.52 |  |
|  | Demonstrate the ability to initiate, participate in, and undertake improvement work as well as the necessary skills for participation in research and development work | 0.51 |  |
|  | Demonstrate advanced ability to discuss new data, phenomena, and issues in the field of medicine on a scientific basis with various audiences as well as critically review, assess, and utilize relevant information | 0.57 |  |
|  | Demonstrate the ability to use digital tools in both healthcare and research and development work | 0.41 |  |
|  | Demonstrate the ability to self-reflect and empathize as well as have a professional attitude | 0.55 |  |
|  | Demonstrate the ability to adopt a health-promoting approach with a holistic view of the patient based on a scientific perspective and with special consideration of ethical principles and human rights | 0.59 |  |
|  | Demonstrate the ability to identify the need for ongoing competence development and to take responsibility for it | 0.56 |  |
| Comprehensibility of the wordings of the scholarly degree objectives^b^ (n=422) | Demonstrate knowledge of the scientific foundation of the field and insight into current research and development work as well as knowledge of the link between science and proven experience in professional practice | 0.40 | 0.84 |
|  | Demonstrate knowledge of fundamental scientific methodology in the field and insight into its opportunities and limitations | 0.53 |  |
|  | Demonstrate knowledge of ethical principles and their application in healthcare and research and development work | 0.62 |  |
|  | Demonstrate knowledge of patient safety, quality, and prioritization in healthcare and methods for evaluating medical practice | 0.59 |  |
|  | Demonstrate the ability to integrate and apply knowledge critically and systematically and analyze and assess complex phenomena, issues, and situations | 0.43 |  |
|  | Demonstrate the ability to initiate, participate in, and undertake improvement work as well as the necessary skills for participation in research and development work | 0.52 |  |
|  | Demonstrate advanced ability to discuss new data, phenomena, and issues in the field of medicine on a scientific basis with various audiences as well as critically review, assess, and utilize relevant information | 0.59 |  |
|  | Demonstrate the ability to use digital tools in both health care and research and development work | 0.46 |  |
|  | Demonstrate the ability to self-reflect and empathize as well as have a professional attitude | 0.56 |  |
|  | Demonstrate the ability to adopt a health-promoting approach with a holistic view of the patient based on a scientific perspective and with special consideration of ethical principles and human rights | 0.56 |  |
|  | Demonstrate the ability to identify the need for ongoing competence development and to take responsibility for it | 0.61 |  |
| Experience of having been trained during the medical program regarding components of a systematic review/HTA^a^ (n=335) | To formulate a research question according to the PICO model | 0.54 | 0.81 |
|  | To find relevant literature according to the PICO model and after literature searches in relevant databases, such as PubMed and the Cochrane Library | 0.62 |  |
|  | To appraise scientific articles using checklists | 0.47 |  |
|  | To synthesize results from several studies, e.g., in a meta-analysis | 0.55 |  |
|  | To assess evidence according to GRADE | 0.61 |  |
|  | To assess organizational aspects related to the introduction or withdrawal of a health technology in healthcare | 0.49 |  |
|  | To assess economic aspects related to the introduction or withdrawal of a health technology in healthcare | 0.43 |  |
|  | To assess ethical aspects related to the introduction or withdrawal of a health technology in healthcare | 0.44 |  |

GRADE = Grading of Recommendations, Assessment, Development, and Evaluations; HTA = health technology assessment; PICO: P = patients, I = intervention, C = comparison, O = outcomes

^a^1 = do not agree at all to 5 = totally agree

^b^1 = “very hard to understand” and 5 = “very easy to understand”
