# Supplementary material for: Preparing medical students to incorporate scientific evidence into patient care: A cross-sectional study
Source: PLoS One. 2025 Apr 4;20(4):e0321211. doi: 10.1371/journal.pone.0321211 (PMC11970701; doi:10.1371/journal.pone.0321211)
Supplement: S5 Table — (DOCX) [file pone.0321211.s006.docx]

**S5 Table.** Multivariate logistic regression analyses to select variables for the final model to identify factors predicting that final-semester students’ agreed with the statement “During the medical program, I have acquired sufficient skills in how to ground patient work on scientific evidence”, i.e., responding 4 or 5 on a scale from 1 = do not agree at all to 5 = totally agree.

|  |  | | **aOR^a^ (95% CI)** | **aOR^b^ (95% CI)** | **aOR^c^ (95% CI)** |
| --- | --- | --- | --- | --- | --- |
| Individual characteristics | Age (≤25 vs. >25 years) | | 1.44 (0.84; 2.46) |  |  |
|  | Sex (female vs. male) | | 1.04 (0.60; 1.80) |  |  |
|  | Worked as a junior physician (yes vs. no) | | **2.01 (1.11; 3.64)** |  |  |
|  | Single best answer questions (≥4 vs. <4 correct answers) | | 1.50 (0.72; 3.11) |  |  |
|  | Master’s thesis was a systematic review (yes vs. no) | | 1.14 (0.42; 3.08) |  |  |
| Experience of the adequacy of the assessment regarding scholarly degree outcomes during the medical program^d^ | Demonstrate knowledge of the scientific foundation of the field and insight into current research and development work as well as knowledge of the link between science and proven experience in professional practice | |  | **6.14 (3.09; 12.2)** |  |
|  | Demonstrate knowledge of fundamental scientific methodology in the field and insight into its opportunities and limitations | |  | 1.25 (0.63; 2.51) |  |
|  | Demonstrate knowledge of ethical principles and their application in health care and research and development work | |  | 0.53 (0.23; 1.22) |  |
|  | Demonstrate knowledge of patient safety, quality, and prioritization in healthcare and methods for evaluating medical practice | |  | 0.83 (0.41; 1.71) |  |
|  | Demonstrate the ability to integrate and apply knowledge critically and systematically, and analyze and assess complex phenomena, issues, and situations | |  | 1.39 (0.70; 2.78) |  |
|  | Demonstrate the ability to initiate, participate in, and undertake improvement work as well as the necessary skills for participation in research and development work | |  | **3.08 (1.57; 6.06)** |  |
|  | Demonstrate advanced ability to discuss new data, phenomena, and issues in the field of medicine on a scientific basis with various audiences as well as critically review, assess, and utilize relevant information | |  | 1.40 (0.72; 2.73) |  |
|  | Demonstrate the ability to use digital tools in both healthcare and research and development work | |  | 1.37 (0.70; 2.69) |  |
|  | Demonstrate the ability to self-reflect and empathize as well as have a professional attitude | |  | 1.74 (0.74; 4.10) |  |
|  | Demonstrate the ability to adopt a health-promoting approach with a holistic view of the patient based on a scientific perspective and with special consideration of ethical principles and human rights | |  | 1.14 (0.55; 2.34) |  |
|  | Demonstrate the ability to identify the need for ongoing competence development and to take responsibility for it | |  | 1.41 (0.71; 2.78) |  |
| Educational content | Experience having undergone education in HTA during the program^e^ | |  |  | **7.68 (1.67; 35.2)** |
|  | Experience of having been trained during the medical program regarding the component in question | To formulate a research question according to the PICO model |  |  | 0.47 (0.17; 1.33) |
|  |  | To find relevant literature according to the PICO model and after literature searches in relevant databases, such as PubMed and the Cochrane Library |  |  | 1.31 (0.46; 3.68) |
|  |  | To appraise scientific articles using checklists |  |  | **3.39 (1.63; 7.06)** |
|  |  | To synthesize results from several studies, e.g., in a meta-analysis |  |  | 1.18 (0.50; 2.77) |
|  |  | To assess evidence according to GRADE |  |  | 1.28 (0.47; 3.48) |
|  |  | To assess organizational aspects related to the introduction or withdrawal of a health technology in healthcare |  |  | **7.84 (2.33; 26.4)** |
|  |  | To assess economic aspects related to the introduction or withdrawal of a health technology in healthcare |  |  | 0.44 (0.16; 1.18) |
|  |  | To assess ethical aspects related to the introduction or withdrawal of a health technology in healthcare |  |  | 1.02 (0.49; 2.12) |
|  | Hands-on, credit-bearing, EBM-related learning activities integrated in clinical courses (yes vs. no) | |  |  | **3.88 (1.50; 10.0)** |
|  | Bachelor and master theses (yes vs. no) | |  |  | 3.07 (0.66; 14.4) |

aOR = adjusted odds ratio; EBM = evidence-based medicine; GRADE = Grading of Recommendations, Assessment, Development, and Evaluations; HTA = health technology assessment; OR = odds ratio; PICO: P = patients, I = intervention, C = comparison, O = outcomes

^a^The model included only variables regarding individual characteristic variables. All variables were a priori included in the final model.

^b^The model included only variables regarding scholarly degree outcome assessments. Statistically significant variables were included in the final model.

^c^The model included only variables regarding educational content. Statistically significant variables were included in the final model.

^d^Agreed (vs. disagreed) to having been adequately assessed on the degree outcome in question or having undergone training regarding the component in question during the medical program: defined as responding 4 or 5 (vs. 1‒3) on a scale from 1= totally disagree to 5 = totally agree

^e^Agreed (vs. disagreed) to having undergone education on HTA: defined as responding 3‒5 (vs. 1‒2) on a scale from 1 = totally disagree to 5 = totally agree (requiring a 4‒5 response was not feasible, as all the responders agreed with the statement in question, i.e., “During the medical program, I developed sufficient skills in how to ground patient work on scientific evidence”)
