# Supplementary material for: Preparing medical students to incorporate scientific evidence into patient care: A cross-sectional study
Source: PLoS One. 2025 Apr 4;20(4):e0321211. doi: 10.1371/journal.pone.0321211 (PMC11970701; doi:10.1371/journal.pone.0321211)
Supplement: S6 Table — (DOCX) [file pone.0321211.s007.docx]

**S6 Table.** Sensitivity regression analyses of all variables in the main analysis and leaving out one university or variable(s) at a time.

|  |  | | **Main analysis aOR (95% CI)** | **Sensitivity analyses, leave-one-out** | | | | | | | | | | |
| --- | --- | --- | --- | --- | --- | --- | --- | --- | --- | --- | --- | --- | --- | --- |
|  |  |  |  | **I (excl GU)** | **II (excl KI)** | **III (excl LiU)** | **IV (excl LU)** | **V (excl UmU)** | **VI (excl ÖU)** | **VII^a^**  **(excl 1 var)** | **VIII^a^**  **(excl 1 var)** | **IX^a^**  **(excl 1 var)** | **X^a^**  **(excl 3 var)** | **X1^b^**  **(excl 2 var)** |
| Individual characteristics | Age (≤25 vs. >25 years) | | 1.51 (0.76; 3.03) | 1.80 (0.75; 4.34) | 1.69 (0.72; 3.96) | 1.34 (0.65; 2.74) | 1.57 (0.77; 3.21) | 1.20 (0.54; 2.65) | 1.61 (0.80; 3.27) | 1.52 (0.76; 3.03) | 1.51 (0.76; 3.02) | 1.52 (0.76; 3.01) | 1.52 (0.77; 3.02) | 1.26  (0.68; 2.34) |
|  | Sex (female vs. male) | | 1.13 (0.58; 2.22) | 1.40 (0.61; 3.24) | 0.65 (0.28; 1.54) | 1.09 (0.54; 2.20) | 1.26 (0.63; 2.49) | 1.43 (0.66; 3.07) | 1.02 (0.52; 2.02) | Excl | 1.13 (0.57; 2.21) | 1.13 (0.57; 2.21) | Excl | 1.02 (0.55; 1.88) |
|  | Worked as a junior physician (yes vs. no) | | 1.61 (0.74; 3.50) | 2.70 (0.91; 8.03) | 1.33 (0.52; 3.40) | 1.44 (0.65; 3.18) | 1.49 (0.67; 3.31) | 2.28 (0.64; 5.51) | 1.34 (0.61; 2.97) | 1.59 (0.73; 3.45) | 1.58 (0.73; 3.42) | 1.61 (0.74; 3.50) | 1.57 (0.73; 3.38) | 1.63 (0.81; 3.26) |
|  | Single best answer questions (≥4 vs. <4 correct answers) | | 1.14 (0.45; 2.87) | 1.88 (0.52; 6.77) | 0.79 (0.27; 2.36) | 0.97 (0.37; 2.51) | 1.47 (0.53; 4.06) | 0.97 (0.36; 2.58) | 1.11 (0.44; 2.84) | 1.13 (0.45; 2.85) | Excl | 1.14 (0.45; 2.85) | Excl | 1.17 (0.52; 2.64) |
|  | Master’s thesis was a systematic review (yes vs. no) | | 0.98 (0.25; 3.92) | 0.27 (0.04;1.84) | 3.51 (0.38; 32.4) | 1.03 (0.26; 4.09) | 0.92 (0.23; 3.70) | 0.93 (0.22; 3.98) | 0.93 (0.23; 3.80) | 1.00 (0.25; 4.02) | 1.00 (0.25; 3.96) | Excl | Excl | 1.75 (0.46; 6.64) |
| Experience of the adequacy of the assessment regarding scholarly degree outcomes during the medical program^c^ | Demonstrate knowledge of the scientific foundation of the field and insight into current research and development work as well as knowledge of the link between science and proven experience in professional practice | | **6.17 (3.10; 12.3)** | **7.75 (3.15; 19.1)** | **8.69 (3.62; 20.9)** | **5.35 (2.64 (10.8)** | **5.92 (2.94; 11.9)** | **6.11 (2.77; 13.5)** | **5.92 (2.95; 11.9)** | **6.17 (3.10; 12.3)** | **6.19 (3.12; 12.3)** | **6.18 (3.13; 12.2)** | **6.21 (3.14; 12.3)** | Excl |
|  | Demonstrate the ability to initiate, participate in, and undertake improvement work as well as demonstrate the necessary skills for participation in research and development work | | 1.76 (0.86; 3.59) | 1.69 (0.70; 4.07) | 1.47 (0.60; 3.56) | 1.93 (0.90; 4.13) | 1.94 (0.93; 4.06) | 1.67 (0.75; 3.75) | 2.27 (1.13; 4.56) | 1.75 (0.85; 3.57) | 1.77 (0.87; 3.61) | 1.75 (0.86; 3.57) | 1.76 (0.86; 3.57) | Excl |
| Educational content | Experience having undergone education on HTA during the program^d^ | | **11.3 (1.44; 89.5)** | 7.57 (0.80; 71.6) | 7.89 (0.95; 65.4) | **10.0 (1.26 (79.3)** | **11.8 (1.49; 93.1)** | NA^d^ | **11.5 (1.46; 90.3)** | **12.6 (1.60; 99.5)** | **11.1 (1.42; 87.2)** | **11.3 (1.44; 89.3)** | **12.4 (1.59; 96.7)** | **14.8 (1.97; 111)** |
|  | Experience of having been trained during the medical program regarding the component in question^b^ | To appraise scientific articles by using a checklist | **2.46 (1.23; 4.90)** | 2.29 (0.96; 5.44) | **2.60 (1.07; 6.32)** | **2.13 (1.04; 4.36)** | **2.41 (1.18; 4.90)** | **3.17 (1.43; 6.99)** | **2.27 (1.13; 4.56)** | **2.45 (1.23; 4.89)** | **2.47 (1.24; 4.93)** | **2.47 (1.24; 4.91)** | **2.47 (1.24; 4.91)** | **2.65 (1.42; 4.95)** |
|  |  | To assess organizational aspects related to the introduction or withdrawal of a health technology in healthcare | **2.65 (1.05; 6.67)** | 3.28 (0.99; 10.8) | 1.60 (0.56; 4.60) | **2.88 (1.079; 7.69** | **2.63 (1.04; 6.65)** | 2.81 (0.94; 8.37) | **2.60 (1.02; 6.64)** | **2.67 (1.06; 6.71)** | **2.64 (1.05; 6.63)** | **2.65 (1.05; 6.65)** | **2.66 (1.06; 6.66)** | **3.31 (1.41; 7.79)** |
|  | Hands-on, credit-bearing, EBM-related learning activities integrated in clinical courses (yes *vs.* no) | | **4.68 (1.69; 13.0)** | **4.52 (1.54; 13.3)** | **5.99 (1.93; 18.6)** | 5.23 (0.995; 27.5). | **4.29 (1.28; 14.4)** | **4.36 (1.50; 12.7)** | **5.02 (1.81; 13.9)** | **4.69 (1.69; 13.0)** | **4.73 (1.73; 13.1)** | **4.72 (1.72; 13.0)** | **4.79 (1.75; 13.1)** | **4.45 (1.76; 11.3)** |

aOR = adjusted odds ratio; EBM = evidence-based medicine; GU = University of Gothenburg; HTA = health technology assessment; KI = Karolinska Institute; LiU = Linköping University; LU = Lund University; NA = not applicable; OR = odds ratio; UmU = Umeå University; var = variable(s); ÖR = Örebro University

^a^Variables were left out if they shifted from >1 in the crude analysis to <1 in the main adjusted model or any sensitivity analysis

^b^Scholarly degree outcomes left out

^c^Agreed (vs. disagreed) to having been adequately assessed on the degree outcome in question, or having had training in the component in question, during the medical program: defined as responding 4 or 5 (vs. 1‒3) on a scale from 1 = totally disagree to 5 = totally agree

^d^Agreed (vs. disagreed) to having undergone teaching in HTA: defined as responding 3‒5 (vs. 1‒2) on a scale from 1 = totally disagree to 5 = totally agree (requiring a 4‒5 response was not feasible, as all the responders agreed regarding the statement in question, i.e., “During the medical program, I have acquired sufficient skills in how to ground patient work on scientific evidence”)
