# Supplementary material for: Preparing medical students to incorporate scientific evidence into patient care: A cross-sectional study
Source: PLoS One. 2025 Apr 4;20(4):e0321211. doi: 10.1371/journal.pone.0321211 (PMC11970701; doi:10.1371/journal.pone.0321211)
Supplement: S1 Data — (DOCX) [file pone.0321211.s008.docx]

**S1 Data.** Details about questionnaire data used in the analyses, by medical program and question.

|  | | | | **Medical program** | | | |
| --- | --- | --- | --- | --- | --- | --- | --- |
|  | **1**  **(n=105)** | **2**  **(n=107)** | **3**  **(n=39)** | | **4**  **(n=95)** | **5**  **(n=28)** | **6**  **(n=59)** |
| Q1a^a^ | 68 | 75 | 33 | | 75 | 19 | 33 |
| Q1b^a^ | 74 | 80 | 36 | | 73 | 24 | 36 |
| Q1c^a^ | 68 | 92 | 35 | | 89 | 20 | 34 |
| Q1d^a^ | 58 | 76 | 32 | | 77 | 18 | 29 |
| Q1e^a^ | 69 | 72 | 34 | | 74 | 18 | 41 |
| Q1f^a^ | 59 | 32 | 26 | | 71 | 15 | 19 |
| Q1g^a^ | 69 | 75 | 32 | | 64 | 23 | 40 |
| Q1h^a^ | 34 | 44 | 24 | | 39 | 15 | 26 |
| Q1i^a^ | 75 | 87 | 37 | | 90 | 24 | 48 |
| Q1j^a^ | 64 | 85 | 29 | | 85 | 17 | 39 |
| Q1k^a^ | 64 | 75 | 32 | | 76 | 20 | 33 |
| Q2a^a^ | 55 | 49 | 23 | | 52 | 14 | 26 |
| Q2b^a^ | 79 | 80 | 30 | | 77 | 25 | 39 |
| Q2c^a^ | 87 | 96 | 35 | | 84 | 25 | 39 |
| Q2d^a^ | 70 | 83 | 35 | | 83 | 24 | 40 |
| Q2e^a^ | 58 | 49 | 25 | | 49 | 15 | 38 |
| Q2f^a^ | 70 | 48 | 20 | | 76 | 19 | 23 |
| Q2g^a^ | 79 | 65 | 28 | | 71 | 24 | 43 |
| Q2h^a^ | 69 | 72 | 26 | | 71 | 19 | 41 |
| Q2i^a^ | 89 | 92 | 36 | | 84 | 25 | 50 |
| Q2j^a^ | 72 | 77 | 30 | | 76 | 19 | 38 |
| Q2k^a^ | 87 | 83 | 33 | | 81 | 23 | 40 |
| Q3^a^ | 81 | 83 | 38 | | 90 | 26 | 41 |
| Q4^b^ | 15 | 24 | 7 | | 29 | 5 | 7 |
| Q5a^a^ | 59 | 18 | 32 | | 68 | 25 | 48 |
| Q5b^a^ | 68 | 28 | 31 | | 69 | 26 | 49 |
| Q5c^a^ | 65 | 81 | 36 | | 67 | 23 | 36 |
| Q5d^a^ | 26 | 25 | 16 | | 59 | 15 | 31 |
| Q5e^a^ | 16 | 9 | 8 | | 52 | 23 | 27 |
| Q6a^a^ | 27 | 28 | 15 | | 42 | 7 | 14 |
| Q6b^a^ | 16 | 23 | 13 | | 24 | 5 | 12 |
| Q6c^a^ | 55 | 69 | 31 | | 76 | 18 | 35 |
| Q7^c^ | 26 | 29 | 12 | | 24 | 8 | 19 |
| Q8^c^ | 98 | 87 | 33 | | 70 | 24 | 49 |
| Q9^c^ | 74 | 72 | 31 | | 74 | 22 | 38 |
| Q10^c^ | 13 | 26 | 8 | | 33 | 6 | 8 |
| Q11^c^ | 52 | 50 | 19 | | 60 | 17 | 24 |
| Q14^d^ | 56 | 64 | 28 | | 57 | 19 | 31 |
| Q15^e^ | 42 | 50 | 23 | | 61 | 14 | 21 |
| Q18^f^ | 11 | 13 | 8 | | 4 | 0 | 0 |
| Q19^g^ | 86 | 68 | 37 | | 81 | 22 | 43 |

^a^Number of students that responded 4 or 5 to the statement in question.

^b^Number of students that responded 4 or 5 to the statement in question.

^c^Number of students that answered correctly.

^d^Number of students that stated female sex.

^e^Number of students ≤25 years of age.

^f^Number of students that stated having conducted a systematic review as research project.

^g^Number of students that stated having worked as an assistant physician.
